# Ion torrent high throughput mitochondrial genome sequencing (HTMGS)

**DOI:** 10.1371/journal.pone.0224847

**Published:** 2019-11-15

**Authors:** N. R. Harvey, C. L. Albury, S. Stuart, M. C. Benton, D. A. Eccles, J. R. Connell, H. G. Sutherland, R. J. N. Allcock, R. A. Lea, L. M. Haupt, L. R. Griffiths

**Affiliations:** 1 Genomics Research Centre, School of Biomedical Sciences, Institute of Health and Biomedical Innovation, Queensland University of Technology, Brisbane, Queensland, Australia; 2 Health Sciences and Medicine faculty, Bond University, Robina, Queensland, Australia; 3 School of Biomedical Sciences, University of Western Australia (M504), Crawley, Western Australia, Australia; University of Helsinki, FINLAND

## Abstract

The implementation and popularity of next generation sequencing (NGS) has led to the development of various rapid whole mitochondrial genome sequencing techniques. We summarise an efficient and cost-effective NGS approach for mitochondrial genomic DNA in humans using the Ion Torrent platform, and further discuss our bioinformatics pipeline for streamlined variant calling. Ion 316 chips were utilised with the Ion Torrent semi-conductor platform Personal Genome Machine (PGM) to perform tandem sequencing of mitochondrial genomes from the core pedigree (n = 315) of the Norfolk Island Health Study. Key improvements from commercial methods focus on the initial PCR step, which currently requires extensive optimisation to ensure the accurate and reproducible elongation of each section of the complete mitochondrial genome. Dual-platform barcodes were incorporated into our protocol thereby extending its potential application onto Illumina-based systems. Our bioinformatics pipeline consists of a modified version of GATK best practices tailored for mitochondrial genomic data. When compared with current commercial methods, our method, termed high throughput mitochondrial genome sequencing (HTMGS), allows high multiplexing of samples and the use of alternate library preparation reagents at a lower cost per sample (~1.7 times) when compared to current commercial methodologies. Our HTMGS methodology also provides robust mitochondrial sequencing data (>450X average coverage) that can be applied and modified to suit various study designs. On average, we were able to identify ~30 variants per sample with 572 variants observed across 315 samples. We have developed a high throughput sequencing and analysis method targeting complete mitochondrial genomes; with the potential to be platform agnostic with analysis options that adhere to current best practices.

## Background

As mitochondria govern the homeostasis of cellular energy requirements, analysis of mitochondrial DNA (mtDNA) variants may provide insight into numerous aspects of inheritance and functionality [1]. Sequencing of highly conserved mtDNA may be useful in heritability studies, various forensic applications, association studies, or the genetic analysis of numerous disease states [2–5]. Although this conservation is relatively consistent for mitochondrial coding sequences, the D-Loop domain spanning the origin of replication consists of three hyper-variable regions (HVI-III) that are routinely studied for heritability [6]. Consequently, forensic identification has previously focussed on these variable regions, although richer information can be gained from identifying mitochondrial haplotypes from complete mtDNA sequencing [6, 7]. Until recently, studies that have shown association with mitochondrial variants have used a limited number (226) of mitochondrial tag SNPs from Genome Wide Association studies (GWAS) or Whole Exome Sequencing (WES) data, rather than the direct analysis of mitochondrial polymorphisms [8, 9]. Whilst the latter would be viable to examine mitochondrial coding regions, it creates an unnecessary bioinformatic workload with higher costs that are not suitable for larger populations.

With an increase in the number of commercially available NGS methods and platforms, the task of performing whole mitochondrial genome sequencing has been made widely accessible. Mitochondrial genome sequencing has the potential to impact a wide variety of health disciplines such as forensic genetics, diagnostics, and complex phenotype association studies. Recent advances in Nanopore sequencing technologies has allowed for sequencing of the mitochondrial genome as a single read, with a minimal calculated error rate of 1% [10]. Key issues for utilising this advanced methodology include the selection of sequencing platform, as well as the selection of a high-quality (accuracy and reproducibility) method for an appropriate price. Currently a variety of kits are available for all platforms including those associated with the instrument manufacturer as well as more affordable “generic” kits (e.g. NEB: Nextfast library prep set; or BIOOscientific: barcodes for Ion Torrent or Illumina applications). As expected, these kits have advantages and disadvantages, which combine to produce a pre-defined end result. Much of the current literature reports on data produced using vendor and platform-specific reagent kits and protocols, with little information available on the successful integration or adaptation of customised library preparation kits within manufacturer sequencing protocols [11, 12].

Sequencing platform selection has been based previously on throughput capabilities, experimental hands-on time, and instrument scalability. Whilst Ion Torrent platforms are commonly associated with ease of scalability, most sequencing platforms are currently on par with this and high sequencing quality output. Our High Throughput Mitochondrial Genome Sequencing (HTMGS) protocol was designed for use on Ion Torrent platforms, but due to the versatility of key reagents, the protocol can also be easily adapted to include Illumina and likely other emerging sequencing platforms.

Experimental success in the NGS era is generally dependent on the throughput capabilities of the instrument, the cost and accuracy per raw base and the length of independent reads [13]. Current commercial methods for mapping and base calling on Ion Torrent platforms commonly involve the use of a server with pre-installed settings for read trimming and quality analysis specific to the instrument that performs the sequencing reaction. The goal of this study was to develop and demonstrate an achieved balance between cost effectiveness and data quality with the flexibility of application to an agnostic platform and subsequent analysis. The methods described in this manuscript support the use of cost-effective reagents and highlight the innovative potential of custom/modified protocols. In addition, the HTMGS protocol provided accurate on-target sequencing data for thorough interrogation of mitochondrial variants and was flexible for data collection and analysis from the wet laboratory component (organism and platform), to analysis approaches. As two established platforms for mitochondrial genomic sequencing are the Illumina HiSeq/MiSeq/NextSeq and Life Technologies/ThermoFisher Scientific Ion Personal Genome Machine (PGM) platforms, this work focussed on protocol development utilising the PGM platform.

## Materials and methods

### Samples

Human blood samples (315) were collected following informed consent as part of the Norfolk Island Health study at the Queensland University of Technology. Genomic and mitochondrial DNA was extracted and purified from whole blood using the QIAamp DNA blood midi kit (Qiagen, Hilden, Germany) according to the manufacturer’s instructions. Ethical clearance for this study was originally provided by the Griffith University Human Research Ethics Committee (Approval Number: MSC/04/09/HREC) and the clearance transferred to and now provided by the QUT Human Research Ethics Committee (Approval Number: 1400000749). For ease of use, the protocol outlined below utilised an Ion Chef instrument (requiring two chips/run for maximum cost effectiveness). The Norfolk Island (NI) population outlined in this manuscript has been extensively studied and included in several publications by our group [14–18]. In an effort to improve data reliability, we have made some minor methodological adjustments to the previously described protocol outlined in the manuscript by Benton M, et al., [17]. We also note that the distribution of mitochondrial haplogroups within our analysis followed the same trend as previously described [17].

### Long range PCR

Initial amplification of targeted mtDNA is most commonly performed using multiple sets of primers. It is generally accepted that shorter products amplify more efficiently than longer products [19], such as in the PrecisionID mitochondrial genome panel supplied by ThermoFisher Scientific. Long range PCR remains the most effective way of limiting the introduction of PCR bias and nuclear contamination into the later sequencing steps. Here we used two long range PCRs with two overlapping primer sets (Fragment 1: mt.569–9819, Fragment 2: mt.9611–626) to generate large products (9,250bp and 8,985bp) suitable for a combined and uniform fragmentation. (Fragment 1 F: 5’-AACCAAACCCCAAAGACACC-3’; Fragment 1 R: 5’-GCCAATAATGACGTGAAGTCC-3’; and Fragment 2 F: 5’-TCCCACTCCTAAACACATCC-3’; Fragment 2 R: 5’-TTTATGGGGTGATGTGAGCC-3’). In addition, this also introduced time and cost advantages over multiple fragment amplification and significantly reduced the inherent complications of nuclear encoded mitochondrial pseudogene amplification [20]. GoTaq (2X) Long PCR Master Mix (Promega, Madison, WI) was used to complete this step. Thermocycling conditions included denaturing: 94.5°C for 2 minutes; cycling: 92 °C for 20 seconds, 60 °C for 20 seconds, 68 °C for 9 minutes; and the final elongation stage: 72 °C for 10 minutes. Fragment 2 amplified more efficiently than fragment 1 with the number of PCR cycles reduced from 35 to 28 for the former to obtain equivalent yields. Interestingly, we found that a low DNA input of 20ng in a 40μl reaction volume significantly enhanced overall product yield compared with the kit-recommended gDNA input of 0.1–0.5μg in a reaction volume of 50μl [21]. Reaction components included 20ng input genomic DNA, 20μl GoTaq Long Master Mix, 200pM of each primer, dH_2_O up to the required 40μl reaction volume. The long range amplification step of the HTMGS protocol may have to be modified for medical diagnostic samples assumed to have a common mt.8470–13447 deletion.

PCR products for both fragments were visualised on 1.0% agarose gels (60V, 60 minutes) with a 1kb ladder (New England Biolabs, Ipswich, MA). Fragments were then purified with QIAquick post PCR clean-up columns from (QIAGEN, Hilden, Germany) and quantified using Agilent DNA 12000 chips on the Bioanalyzer 2100 system (Agilent Technologies, Santa Clara, CA). The highest concentrated fragment (F1 or F2) of each corresponding sample was diluted to match the lowest concentrated fragment (F1 or F2) and then pooled at equimolar concentrations to a final amount of 100ng in 51μl. This step ensured an even representation of the fragmented mtDNA for optimised and consistent coverage of the complete genome.

### Physical fragmentation and end-repair methods

To add to ease and cost effectiveness of the protocol, we utilised physical shearing with sonication using the Bioruptor system (Diagenode, London) due to its selective design for small sample volumes and effectiveness in maintaining sample integrity. Considering our desired fragmentation profile (50–500bp, peak at 200bp), 200ng of pooled mtDNA in a final volume of 51μl was sheared using the following parameters: 5x (15 cycles of 30secs high intensity sonication followed by 30secs off). All 200ng of fragmented mtDNA was end repaired using the NEB end repair module kit (New England Biolabs, Ipswich, MA) according to the manufacturer’s instructions.

### Barcoding and size selecting methods

Ion Torrent compatible-NEXTflex DNA Barcodes- (Bioo Scientific, Austin, TX), were selected as a cost-effective alternative to standard IonXpress barcodes supplied by ThermoFisher Scientific. Barcode ligation was performed with the T4 DNA ligase enzyme component of the NEBNext fast DNA library prep set for Ion Torrent (New England Biolabs, Ipswich, MA). Barcoded products were then subjected to a purification process using 180μl Agencourt AMPure XP mix (Beckman Coulter, Brea, CA) and subsequent 80% (v/v) Ethanol wash. Following this, the barcoded mtDNA fragments were examined on an E-gel system (ThermoFisher Scientific, Waltham, MA) utilising an iBase unit to visualise the running gel. This allowed us to identify and recover fragments between 250–350 base pairs. Libraries were size selected at a target peak of 330bp thus maximising efficiency under the limitations of 200bp sequencing chemistry.

### Library amplification of mtDNA

Size selected amplicons underwent final library amplification using the NEBNext Fast DNA Library Prep Set for Ion Torrent (New England Biolabs, Ipswich, MA). We found that 100ng of input mtDNA combined with the manufacturer defined protocol aided in high quality library generation. Amplified libraries were purified using 140μl Agencourt AMPure XP magnetic beads and a subsequent 80% (v/v) Ethanol wash. Purified products were quantified using Bioanalyzer system with Agilent DNA 1000 chips (Agilent Technologies, Santa Clara, CA).

### Template preparation and semiconductor sequencing

For templating, all 315 mitochondrial libraries were diluted to achieve a final equimolar concentration of 25pM. To achieve maximum efficiency, we pooled between 35 and 49 mitochondrial libraries for each sequencing chip. The libraries then underwent template preparation using an Ion Hi-Q View Chef Kit (ThermoFisher Scientific, Waltham, MA) on an automated Ion chef system (ThermoFisher Scientific, Waltham, MA). The mitochondrial libraries were loaded onto Ion 316 V2 BC chips (ThermoFisher Scientific, Waltham, MA). We ran two chips at once to prevent reagent waste from the Ion Chef and sequencing kits. Semiconductor sequencing was performed using an Ion PGM Hi-Q view Sequencing 200 Kit (ThermoFisher Scientific, Waltham, MA) on an Ion Torrent PGM platform (ThermoFisher Scientific, Waltham, MA). All protocols followed standard manufacturer’s instructions. Following successful amplification and sequencing of mitochondrial libraries, read trimming, base calling and mapping (Revised Cambridge Reference Sequence [rCRS]) was completed using the Ion Torrent Suite software V4.4.3 (ThermoFisher Scientific, Waltham, MA USA). It should be noted that the cost comparison of methodologies shown in Table 1 used current prices as of November 2018 for reagents.

**Table 1:** Quality metric comparison between suggested commercial sequencing quality and results from both HTMGS sequencing chips.

Chip number	1	2	3	4	5	6	7
**Chip loading (%)**	65	82	77	80	80	61	78
**Enrichment (%)**	100	100	100	100	100	100	100
**Polyclonal (%)**	18	45	45	42	44	19	47
**Low quality (%)**	3	23	22	11	15	4	15
**Primer Dimer (%)**	1	0	0	1	0	5	0
Usable reads* (%)	3,182,960 (79.0)	2,190,789 (42)	2,040,115 (42)	2,550,966 (51)	2,415,427 (48)	2,839,445 (73)	2,206,387 (45)
**Mapped usable reads (%)**	99.7	97	97	96	97	98	96
**Mean Read length (bp)**	126	159	185	182	180	125	147
**Total bases (>AQ17)**	374Mb	303Mb	292Mb	371Mb	355Mb	331Mb	253Mb

*This value is dependent on the overall sequencing quality for the rest of the measurements

### Variant curation and haplotype calling

Raw fastq files were exported from the Ion Torrent PGM Server, processed through FastQC [22] and a unified sequencing quality report created using multiQC [23]. Further analysis was performed using Linux based bioinformatics programs (*SAMtools and BCFtools version 1*.*6 (htslib 1*.*6-56-glc5c508)*, *tabix*, and *GenomeAnalysisTK* (*GATK) v3*.*8* for use with *FastaAlternateReferenceMaker*) [24–26]. While there are updated human reference mitochondrial genomes, the majority of annotation tools utilise the rCRS reference genome [27]. Therefore, we mapped the fastq files to the rCRS using a Burrows-Wheeler alignment (*BWA mem*, *version 0*.*7*.*17-r1188*). Variant calling was performed using SAMtools *mpileup* and *BCFtools norm* was used to normalise and left align the sequences to the rCRS reference genome and account for the limitations of single end sequencing. The files were then converted to VCF format and indexed with *tabix*. By looping all samples through the *GATK FastaAlternateReferenceMaker*, individual fasta files for each sample were generated. A merged VCF file was created using *vcf-merge* and a merged fasta file was generated by concatenating all samples. The merged fasta file was utilised for the online tool Mitomaster to annotate all variants and call haplotype information for each sample [28]. The merged VCF file was uploaded to the online tool haplogrep2 to ascertain the confidence level of variant calls and generate a phylogenetic tree with all mitochondrial haplogroups seen in the population [29]. The code utilised for this study is available in the GitHub repository https://github.com/GRC-CompGen/mitochondrial_seq_pipeline. A custom R script was used to create a solar plot of variant frequencies observed in the samples sequenced.

## Results

Output sequencing produced approximately 2.5x10^6^ of 200bp-single-end reads per Ion 316 chip. The mean sequencing depth across all 315 samples was 464X (average reads per sample was 58,883, SD±12,171) (Table 1). All seven sequencing chips had comparable data outputs and generated files with equivalent coverage over the same density of the mitochondrial genome. It is important to note that due to a mixture of high polyclonal hits and low chip loading (chips 1 and 6); capacity was reduced by approximately 50% for all chips.

**Fig 1:**
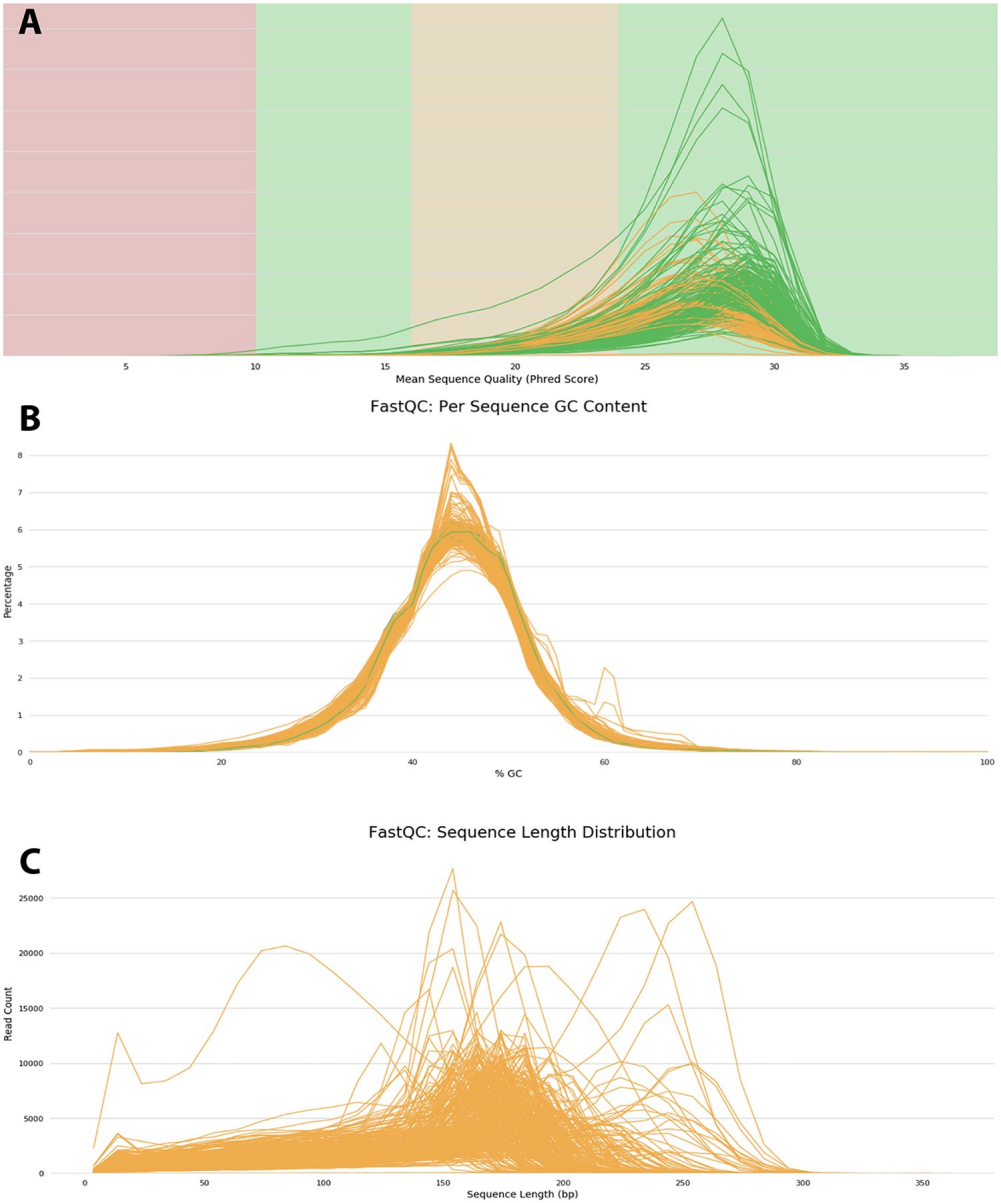
Summary of quality control metrics for the reads generated by the HTMGS protocol by MultiQC. A) Phred quality scores remained consistent per base in read when considering size selection methods; B) sequence GC content followed a normal distribution. The green line represents ‘excellent’ quality reads whereas orange lines represent ‘medium’ read quality scores; C) read length distribution for all samples was between 150–250bp due to the size selection method. The orange lines represent ‘medium’ read quality scores.

Sequence quality (Phred) scores remained consistent at >25 for all samples at the median read length (200bp) (Fig 1A) and per sequence GC content followed a normal distribution as expected for NGS data (Fig 1B). Sequence length distribution was inconsistent which was expected given the size selection method utilised for this study (Fig 1C). The median GC content quality scores were as expected with sequencing of a specific area yielding a larger number of duplicate reads. The medium read length quality scores were due to inconsistent (150–250bp) read lengths from a combination of the shearing method, size selection method (Egel selection for 200bp), and the variable length sequencing typical of Ion Torrent chemistries.

**Fig 2:**
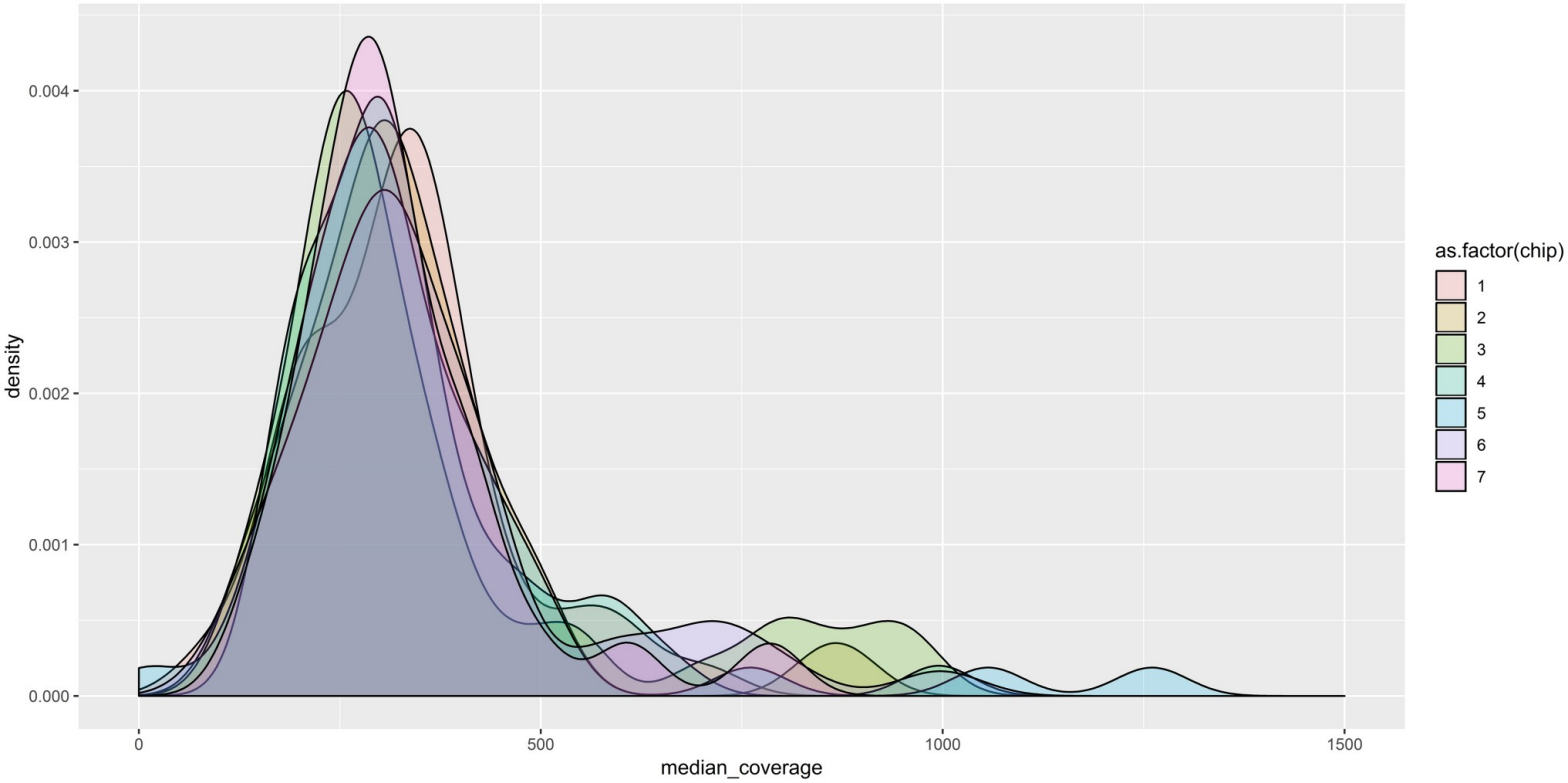
Median coverage density plot for all 315 HTMGS runs showing consistent median coverage between all sequencing chips.

Although the data indicated that we utilised only 50% of the chip capacity on average, high coverage (>250X) per sequencing reaction was generated. To determine the median sequencing coverage across the mitochondrial genome, we generated a median coverage density plot (Fig 2) with our HTMGS method reaching a median depth of ~350X for all 315 samples.

**Fig 3:**
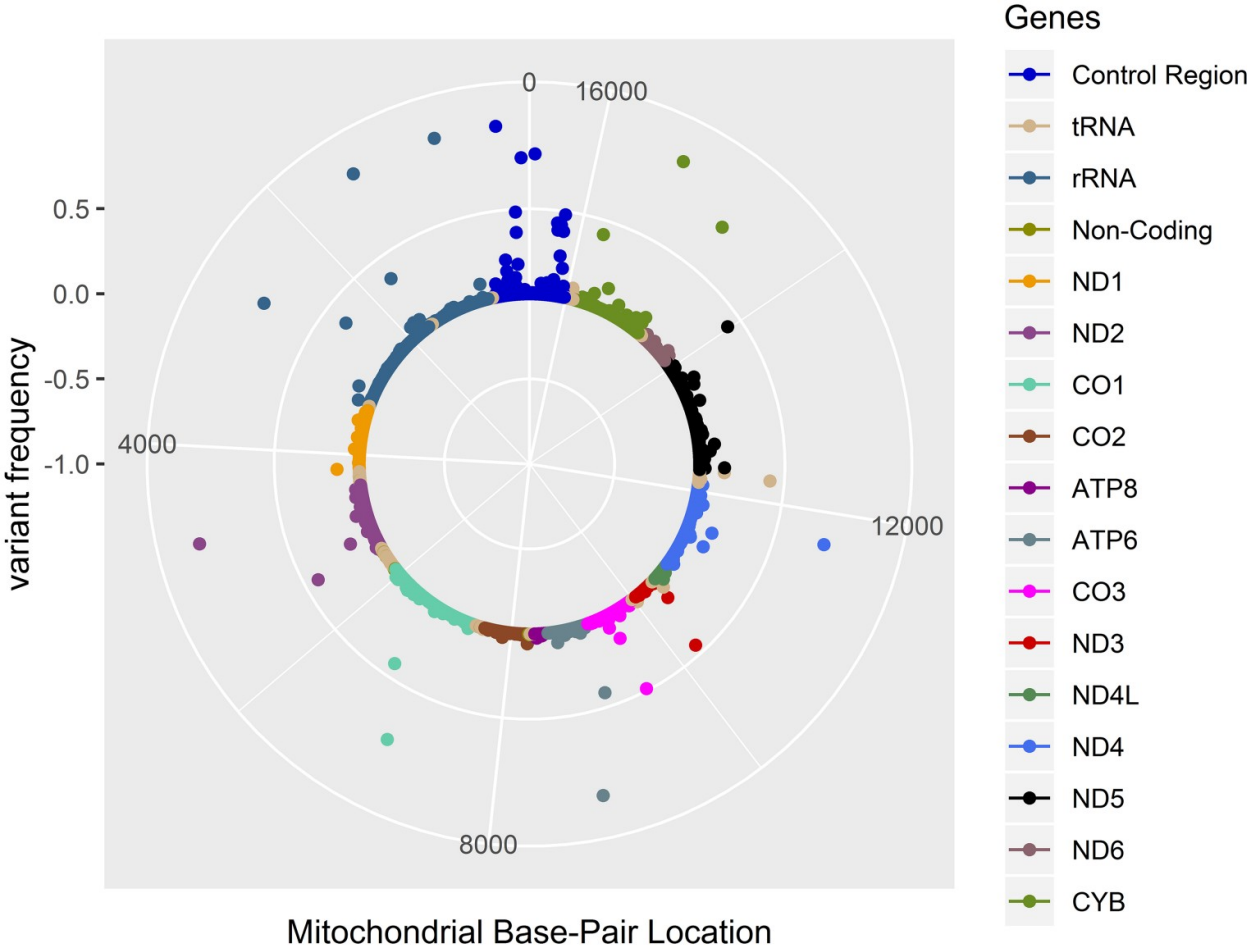
Evidence of correct variant calling in the HTMGS method via examination of mtDNA variant distribution and concordance. The solar plot summarises the variants detected via the HTMGS protocol. Each dot represents a detected variant. The inner ring of the plot represents the mitochondrial genome and is coloured based on genomic region as summarised in the plot legend. The X-axis represents the mitochondrial base pair location with the Y-axis representing the variant frequency in the NI population from 0 (not present in the NI sample) to 1 (total concordance of the variant in the NI sample). The location of the data points in relation to the inner ring represents the allele count with those on the centre ring correlating to 1/315 in the NI pedigree whereas variants toward the outer ring varying and those touching the outer ring representing total concordance of the variant in the NI sample (315/315).

The HTMGS method appears to have resolutely sequenced much of the mitochondrial genome with a high quality sequencing depth (>100X) across all seven chips. Although some samples (n = 39) reached a median coverage >500X, the majority of the reads (59%) clustered between 250X and 500X coverage. Variants were annotated via the Mitomaster online software and a list of variant frequencies was produced based on the output file. The frequencies and locations of the variants were plotted in R using a custom script (Fig 3) [30]. Haplogrep2 was used to determine the confidence levels of all variants prior to plotting.

Approximately one third of total variants (n = 202/571) had a variant allele count of one (i.e. only present in one individual) in the entire population. These were represented by the inner ring of data points in Fig 3. The other 369 variants were identified to be shared between at least two participants as shown by the data points emerging from the inner ring of ‘singleton’ variants. None of the variants identified were observed to be 100% concordant in the population with the largest frequency of n = 314/315 allele counts for the g.A8860G variant within the *ATP6* gene. Given the nature of this cohort (genetic isolate) a large amount of overlap of variants seen across the participants was expected.

## Discussion

### Sequencing

NGS has made whole mitochondrial genome sequencing widely accessible for applications such as diagnostics and research of complex disorders. As NGS technologies continue to be developed, the benefits of high throughput, high quality, and low-cost methodologies continues to rise. With more accurate optimisation of the sequencing protocol and updated sequencing reaction kits, we estimated that the coverage metrics developed in the HTMGS protocol would double for each of the 48 samples placed on our Ion 316 chips. This was demonstrated in Table 2, where comparable chip loading and enrichment measurements were shown across all seven Ion 316 chip sequencing reactions.

**Table 2:** Commercial kit verses HTMGS method cost comparisons for each major step used to complete Ion Torrent PGM whole mitochondrial sequencing.

**HTMGS method**	**Kit**	**Cost per sample (AUD)**
Long range PCR	GoTaq long PCR Master Mix	$2.45
Cleaning and pooling	Qiaquick spin columns	$1.94
	DNA 12000 Bioanalyser kit	$3.62
	50bp ladder	$0.16
Size selection	Egel (2% size select)	$3.85
Library Preparation	Next fast library prep set for ion torrent	$16.80
	Bioo Scientific barcodes (96)	$3.34
	AMPure XP beads	$7.86
	DNA 1000 Bioanalyser kit	$3.62
	**Total cost**	**$43.64**
**Commercial kit**	**Kit**	**Cost per sample (AUD)**
Long range PCR	**Expand long range dNTP pack (125)**	**$2.08**
Cleaning and pooling	Qiaquick spin columns (100)	$1.94
	DNA 12000 Bioanalyser kit	$3.62
	50bp ladder	$0.16
Size Selection	Egel (2% size select)	$3.85
Library Preparation	**IonXpress 1–96 barcodes**	**$12.10**
	**Ion plus fragment library kit**	**$43.60**
	AMPure XP beads	$7.86
	DNA 1000	$3.62
	**Total cost**	**$78.83**

All costs shown are current as of November 2018. All materials and prices shown in bold differ from the HTMGS protocol and other reagents were assumed to be comparable.

In addition, as depicted in Table 2, the total cost of complete mtDNA NGS sequencing was 1.7x cheaper when using the HTMGS method when compared to the same method using commercial, platform specific kits encompassing all reaction components (HTMGS: AUD ~$45/sample, Commercial: AUD ~$80/sample). The significant cost reduction of approximately 50% per sample combined with the high-quality sequencing output further supports the HTMGS method as an ideal alternative to current commercial methods. We note that the long range PCR kit used in the final HTMGS method was marginally more expensive than the equivalent kit supplied by Roche as previously described in Benton et al., [17]). However, the combination of availability, consistency, and price of the GoTaq Promega kit led to improved overall cost effectiveness, due in part to the reduced need for repeat PCRs. Similar applications for alternate library preparation reagents may be expanded to include Illumina based sequencing methodologies, primarily due to the Biooscientific barcode sequences, which contain Illumina barcode sequences. Although these have previously not offered the ease of scalability comparable to the Ion platforms, both platforms are currently on par with regard to scalability and ease of use [31]. The additional amplicon cleaning step performed after the long-range PCR in the HTMGS method is thought to have contributed to the increase in library quality in proceeding steps. The selection of the GoTaq Long Range Master Mix reagent incurred an increased reaction cost per sample when compared to other enzymes such as the Roche/Merck Millipore Expand dNTP long-range kit (Table 2: HTMGS: AUS $2.45/sample, Commercial: AUS $2.08/sample). This kit was selected as the preferred long-range template kit due to an observed consistent rate of reproducibility. In addition, the library preparation cost/sample was substantially reduced in our method when compared to kits from Life Technologies.

### Analysis

Ion Torrent technology has previously been problematic for high sequencing error rates and homopolymer calling issues. Updated sequencing chemistry and bioinformatics pipelines tailored to single end reads have negated this issue. While there is bioinformatics software inbuilt into the Ion Torrent platform, we aimed to develop a truly agnostic approach that uniquely mapped mitochondrial sequencing reads to the correct location. To achieve this goal, we developed a bioinformatics pipeline tailored to variant calling for mitochondrial data which may be adapted to various sequencing outputs (i.e. Ion Torrent/Proton, Illumina). Our pipeline incorporates key changes (outlined below) that allow for the agnostic approach.

Like other NGS analysis pipelines, our custom pipeline initially mapped the unmapped reads to a reference genome using the *BWA MEM* algorithm instead of the *TMAP* open source software used by the Ion Torrent Suite software. As we solely utilised BWA as an agnostic alternative mapping tool, we did not perform comparisons between the two programs. We utilised the SAMtools mpileup function and BCFtools call function to normalise and call variants and INDELS. The *PICARD* program utilises the Java High Throughput Sequencing (HTS) library from *SAMtools* to filter duplicate reads and; as we already utilised *SAMtools*; justified our use of the program in the pipeline. For the single end reads used in our sequencing data, the use of *CIGAR* to recover clipped 5`ends of reverse strand reads was unnecessary. The alignment of INDELs prevented the discordance of variant calling in downstream processes. In using the *BCFtools* function in the *SAMtools* package, we ensured consistency of the programming language and ease of use in our pipeline whilst maintaining appropriate INDEL calling and realignment. Read quality and Phred scores were analysed using FastQC in our method compared with BaseRecalibrator in the GATK suggested method. The *BCFtools norm* function was used to remove duplicate reads, call variants, and recalibrate the realigned bam files firstly according to SNPs and then to account for INDELS. Using one program for the above steps ensured streamlined processes for large amounts of mitochondrial genome samples.

The variant frequencies, as depicted by the solar plot in Fig 3, remained consistent for all mitochondrial genes except for the aptly named hypervariable region. As all mitochondrial coding regions are important to maintain cell function any variation in the mitochondrial genome potentially contributes to mitochondrial associated disease traits. We expected a greater number of SNPs in the hypervariable region as this was consistent with current literature. Apart from these regions, variant frequencies were consistent across the genome with the majority of variants in no more than 20 samples.

## Conclusions

Our modified HTMGS protocol provided comparable quality sequencing data and mitochondrial genome coverage/sample compared to the manufacturer’s recommended output and demonstrated increased value for money. We assessed components of the protocol including the total cost of the library preparation reagents used in each protocol with their respective reaction amounts to determine the cost of reagents on a per sample basis. Additionally, as sequencing reagent costs are relatively similar, the increased multiplexing capability of samples (n = 48) shown in our protocol should further increase the price gap between the methodologies. It should also be noted that the use of an Ion Chef instrument for templating in our method likely contributed to our observed high yield and sequencing quality.

This method may be further adjusted for higher throughput capabilities, which will decrease coverage per sample and deliver a reduced cost per sample for sequencing. We estimate that it will be possible to multiplex n = 96 samples onto an Ion 316 chip for an approximate coverage of 200X per sample (assuming chip utilisation at full capacity). For studies that require large amounts of samples and an increased coverage depth we recommend utilising the scaling capabilities of the Ion torrent instruments and the capacity of other available chips.

The methods developed as part of this study have proven to be more cost effective than those currently available without compromising the accuracy of the sequencing data and these methods could have significant applications to diagnostics, forensics, and analysis of other mitochondrial genomes. For forensic and diagnostic applications, coverage of the mitochondrial genome is not strictly specified and is more stringent on the concomitant replication of identification results via Sanger sequencing (Scientific Working Group on DNA Analysis Methods [SWGDAM]/National Association of Testing Authorities [NATA] guidelines). Multiple studies examining the use of NGS for medical diagnostic testing have specified that a coverage depth of >1000X is sufficient to reliably call mtDNA variants at low heteroplasmy [32–34]. Further optimisation of total coverage should be performed when considering heteroplasmic associations.

We have developed and evaluated a new NGS mitochondrial sequencing protocol for whole mitochondrial genome sequencing. The HTMGS protocol utilises multiplexing of samples onto the same Ion chip and delivers comparable high quality results whilst being more cost effective than current commercial methodologies.

## Abbreviations

BWA: Burrows-Wheeler alignment
dNTP: Deoxynucleotide Triphosphate
GATK: Genome Analysis TK
GRC: Genomics Research Centre
GWAS: Genome Wide Association studies
HTMGS: High Throughput Mitochondrial Genome Sequencing
HTS: Thigh Throughput Sequencing
HVI-III: Hyper-variable regions I-III
mtDNA: Mitochondrial DNA
NATA: National Association of Testing Authorities
NCRIS: National Collaborative Research Infrastructure Strategy
NGS: Next Generation Sequencing
NHMRC: Australian National Health and Medical Research Council
NI: Norfolk Island
OT2: One Touch 2
PGM: Personal Genome Machine
rCRS: revised Cambridge Reference Sequence
RSRS: Reconstructed Sapiens Reference Sequence
SWGDAM: Scientific Working Group on DNA Analysis Methods
WES: Whole Exome Sequencing
